# The role of interpersonal communication in the process of knowledge mobilization within a community-based organization: a network analysis

**DOI:** 10.1186/1748-5908-9-59

**Published:** 2014-05-22

**Authors:** Heather L Gainforth, Amy E Latimer-Cheung, Peter Athanasopoulos, Spencer Moore, Kathleen A Martin Ginis

**Affiliations:** 1 School of Kinesiology and Health Studies, 28 Division Street, Queen’s University, Kingston, ON K7L 3N6, Canada; 2 Spinal Cord Injury Ontario, 520 Sutherland Drive, Toronto, ON, M4G 3V9, Canada; 3 Department of Kinesiology, McMaster University, 1280 Main St. West, Hamilton, ON L8S 4K1, Canada

## Abstract

**Background:**

Diffusion of innovations theory has been widely used to explain knowledge mobilization of research findings. This theory posits that individuals who are more interpersonally connected within an organization may be more likely to adopt an innovation (*e.g.*, research evidence) than individuals who are less interconnected. Research examining this tenet of diffusion of innovations theory in the knowledge mobilization literature is limited. The purpose of the present study was to use network analysis to examine the role of interpersonal communication in the adoption and mobilization of the physical activity guidelines for people with spinal cord injury (SCI) among staff in a community-based organization (CBO).

**Methods:**

The study used a cross-sectional, whole-network design. In total, 56 staff completed the network survey. Adoption of the guidelines was assessed using Rogers’ innovation-decision process and interpersonal communication was assessed using an online network instrument.

**Results:**

The patterns of densities observed within the network were indicative of a core-periphery structure revealing that interpersonal communication was greater within the core than between the core and periphery and within the periphery. Membership in the core, as opposed to membership in the periphery, was associated with greater knowledge of the evidence-based physical activity resources available and engagement in physical activity promotion behaviours (ps < 0.05). Greater in-degree centrality was associated with adoption of evidence-based behaviours (p < 0.05).

**Conclusions:**

Findings suggest that interpersonal communication is associated with knowledge mobilization and highlight how the network structure could be improved for further dissemination efforts. Keywords: diffusion of innovations; network analysis; community-based organization; knowledge mobilization; knowledge translation, interpersonal communication.

## Background

Knowledge mobilization—the act of moving research results into the hands of research users—has become an emerging priority among academic communities and funding agencies [1,2]. Knowledge mobilization ensures that the resources and the time that have been devoted to conducting research are not wasted and that effective evidence-based interventions and policies are accessible to the general population [3,4]. Despite its importance, mobilization of research findings is often slow or non-existent. There is limited understanding of how to ensure research is used in practice [5-8]. While research has examined various strategies to encourage knowledge mobilization, few studies have acknowledged or examined the underlying complex process of mobilizing research evidence [7-9]. In particular, there is inadequate understanding of how communication networks (*e.g.*, who is in contact with whom) account for the success or failure of knowledge mobilization efforts [7].

Diffusion of innovations theory has been widely used by researchers to begin to understand the mobilization of research findings in healthcare and public health settings [10-12]. This theory seeks to explain how new ideas and practices (*e.g.*, research findings) spread between and within social systems (*e.g.*, research users in organizations). According to the tenets of diffusion of innovations theory, the process of knowledge mobilization is inherently social. The relationships between individuals in an organization and the overall communication structure of these relationships can affect the extent to which research findings will be adopted [13]. Rogers posits that individuals who are more interpersonally connected within a social system may be more likely to adopt an innovation than individuals who are less interconnected within the system [13]. Despite Rogers’ assertion that interpersonal communication may be pivotal for the process of knowledge mobilization to occur, research examining the nature of interpersonal communication in knowledge mobilization is limited.

Network analysis is an empirical approach to examining how the overall pattern of interpersonal communication within an organization affects the process of knowledge mobilization. Network analysis provides a valuable set of theories, tools and methods for describing, exploring and understanding the structural and relational aspects of a group [14]. Using network analysis, insight can be gained into the pattern of interpersonal communication existing among individuals within an organization, and how these patterns may influence the adoption of research findings among these individuals [14].

Network analysis research has demonstrated that social network properties can affect individual adoption of health related behaviours and medical innovations [15-18]. Network analysis research has also been used to understand how individuals within an organization share evidence-based information [19,20]. However, neither of these studies specifically examined the relationship between network structure and the adoption of evidence-based practice (*i.e.*, whether research evidence was taken up and used). Valente *et al.*[21] and Fujimoto *et al.*[22] conducted a longitudinal network analysis of community leaders working within community coalitions to examine the association between network structure and the adoption of an evidence-based substance abuse prevention program. Results of both studies were contrary to Rogers’ diffusion of innovations theory which predicts that dense (*i.e.,* more pathways for communication) and centralized (*i.e.,* pathways of communication directed at 1 or a few members) networks should facilitate the adoption of evidence-based programming. Valente *et al.*[21] found that increased adoption of evidence-based practices was associated with coalition networks’ density decreasing over time. Fujimoto *et al.*[22] showed using the same data that adoption of evidence-based practice was dependent on the nature of the relationship being examined. Adoption through advice seeking was associated with less centralized networks whereas adoption through discussion was associated with more centralized networks. The broad findings from these investigations point to an association between network properties and knowledge mobilization. Thus, findings are contrary to diffusion of innovations theory and highlight that we should not assume that dense interpersonal communication is associated with evidence-based practice in every setting [21].

Similar to the work of Fuijimoto *et al.* and Valente *et al.*[21,22], the present study aims to examine the role of network structure and interpersonal communication in knowledge mobilization. Our study is unique in that we use a whole network design (*i.e.,* respondents can be linked to one another) to examine the overall structure of knowledge mobilization within the novel context of a community-based organization (CBO). A CBO is a not-for-profit organization that has a mandate to provide programs and services to members of their community are often marginalized and/or stigmatized members of societies (*e.g.*, persons with disabilities) [23]. In knowledge mobilization, CBOs are important and strategic organizations to examine because they act as key intermediaries between researchers and the marginalized communities served by CBOs [12,13,23-25]. In the present study, we examined the adoption of new evidence-based physical activity guidelines for people with spinal cord injury (SCI) among the staff of a CBO that assists people with SCI and other physical disabilities [25]. In particular, we aimed to determine whether exposure to interpersonal communication about physical activity was associated with adoption of the guidelines by staff. Adoption was defined as having knowledge of the evidence-based physical activity resources available and engaging in physical activity promotion behaviours.

Consistent with Valente *et al.*[21] and Fujimoto *et al.*[22], we examined the association among the density of the CBO network, the centrality of staff in the network, and their adoption of the guidelines. The association between density and adoption was assessed by examining the core-periphery structure of the network which consists of a core group of actors who are densely connected to one another (the core) and a separate group of actors that are loosely (or not at all) connected to the core. Consistent with diffusion of innovations theory, we hypothesized that as opposed to individuals on the margins of the network (*i.e.,* periphery), core individuals would be more likely to have greater knowledge of the evidence-based physical activity resources available and engage in physical activity promotion behaviours. To assess the relative prominence of actors within the CBO network, degree centrality was assessed. This measure represents the extent to which actors are connected to all the other actors in a network, and reflects the number of ties an actor either sends to or receives from other network actors [26]. Consistent with our previous hypothesis, we expected that degree centrality would be associated with greater knowledge of the evidence-based physical activity resources available and engagement in physical activity promotion behaviours.

## Method

The methodology and participant demographics have been previously described (Gainforth HL, Latimer-Cheung AE, Athanasopoulous P, Moore S, Martin Ginis K: Using Network Analysis to Understand Knowledge Mobilization in a Community-based Organization, Submitted). Only a procedural overview is provided below.

### Study design

The study used a cross-sectional design to evaluate the CBO’s knowledge mobilization network. Given that the organization is a small and bounded collective, the network was evaluated using a whole network design [27]. The CBO’s roster of staff within the organization was used to identify and set the network boundary [28]. The present study investigated all of the relations between staff (n *=* 78) who work within the service provision branch of the organization^a^. These individuals work to assist clients of the CBO who have a SCI or a physical disability in the transition from acute care through rehabilitation and back into the community. The focal point of the network was the relationship between individuals exchanging information or sharing resources to advance physical activity knowledge and participation among Canadians living with SCI. The study was approved by the Queen’s University General Research Ethics Board.

### Questionnaires

#### Network instrument

At the time the network analysis was conducted, knowledge mobilization activities had been occurring within the CBO for seven months. Therefore, participants were asked about sharing information about physical activity for people with SCI in the last seven months. Sharing information about physical activity for people with SCI was specifically defined as receiving information and/or providing information about physical activity for people with SCI. To maintain clarity, the online network instrument was divided into four sections: clients; people within the CBO; people outside of the CBO; and resources.

The first section pertaining to how information about physical activity was shared with clients had three questions. Participants indicated the number of clients that they had spoken to about physical activity in the last seven months; had asked them about physical activity in the last seven months; and they had worked with in the last seven months. Participants were specifically told to indicate only frequencies and not the names of clients to maintain client anonymity. In the second and third section, participants were allowed to use names and freely recalled the names of people within and outside of the CBO with whom they had shared information about physical activity in the last seven months. Except for client names, participants were free to name as many people as they wished by inputting individuals’ names into the online network instrument.

### Adoption of physical activity promotion resources and behaviour

To assess the staff’s adoption of physical activity promotion resources and behaviour, we assessed their knowledge of the physical activity promotion resources offered to staff as well as their engagement in activities that are indicative of a choice to adopt or reject physical activity promotion [13]. To assess knowledge, participants indicated whether they had heard of the physical activity guidelines for people with SCI and SCI Action Canada (yes/no response).

To assess behaviour, participants responded to a series yes or no questions adapted from Cameron *et al.*[29] about engaging in activities indicative of promotion physical activity using the stem ‘over the past seven months….’ Behaviours included visiting the SCI Action Canada website; speaking to an individual with SCI about physical activity; and downloading a copy of the physical activity guidelines for people with SCI [30]. Of note, SCI Action Canada is a group of researchers and community members who aim to develop and mobilize strategies to inform, teach and enable people living with SCI to maintain a physically active lifestyle. The SCI Action Canada website offers several evidence-based physical activity resources for people with SCI including the guidelines (see http://www.sciactioncanada.ca). A principal component analysis revealed a one-factor solution for the three behaviour items (*i.e.*, visiting the SCI Action Canada website; speaking to an individual with SCI about physical activity; and downloading guidelines). The Kaiser–Meyer–Olkin measure verified adequate sampling for the analysis, KMO = 0.62 [31]. The single factor had an eigenvalue greater 1 (*=* 2.06) which explained 68.56% of the variance. Therefore, the behaviour items were summed to create a scale (Cronbach’s alpha = 0.77). Higher scores on this combined scale indicate greater adoption of physical activity promotion behaviours.

### Analysis plan

Network data were analyzed using a one mode network design. All relationships between the staff were examined based on their information sharing. The network analysis was performed using UCINET v6 [32] and NETDRAW [33] software. Because we were interested in the degree to which staff might send or receive information to others, we examined our network data as an undirected, symetric network.

To identify potential covariates, we conducted a series of chi square tests of independence on categorical demographic variables (*i.e.*, sex, education, SCI) and Analysis of Variance (ANOVAs) on continuous demographic variables (*i.e.*, years worked for the CBO, age). To assess whether membership in the core or the periphery was associated with knowledge of the evidence-based resources, we conducted a series of 2 (Interpersonal Communication: core versus periphery) × 2 (Knowledge: yes versus no) chi square tests of independence. If the expected cell count was less than 5, Fischer’s Exact Test was used. ANOVAs were conducted to assess whether membership in the core or the periphery was associated with staff engaging in knowledge mobilization. To assess whether degree centrality was related to knowledge mobilization, biserial correlations examining the relationship between in and out degree centrality measures and adoption indicators were conducted. To account for the inherent non-independence of network data, all tests were bootstrapped (Samples = 1,000).

## Results

### Participants and general network description

**Figure 1:**
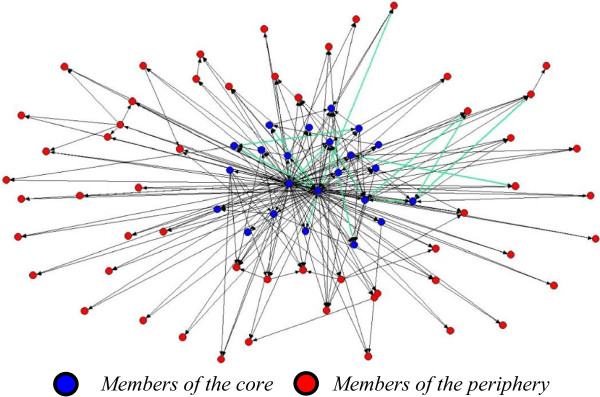
**CBO network structure.** Note. Green lines denote reciprocal ties.

A total of 56 staff (M_age_ = 48.61; SD = 39.80 yrs) completed the network survey (72% response rate). Staff were predominantly female (77%) and university educated (72%). On average, staff worked for the CBO for 4.64 years (SD = 4.94) and the majority of staff did not have a SCI (77%). In total, participants named 78 staff with whom they shared information and 243 ties were reported. The network had a density of 4% and reciprocity of 8%. The patterns of densities within the network were indicative of a core-periphery structure (see Figure 1). The density of ties among the core actors was 16%; the density of ties sharing information from the core to the periphery was 10%; the density sharing information from the periphery to the core was 0.1%; and the density of ties sharing information among periphery actors was 0.1% (Test Fitness = 0.33). Demographic characteristics were similar across groups (ps >0.05). Additional descriptive information on the CBO network can be found elsewhere (Gainforth HL, Latimer-Cheung AE, Athanasopoulous P, Moore S, Martin Ginis K: Using Network Analysis to Understand Knowledge Mobilization in a Community-based Organization, Submitted).

### Core-periphery structure and knowledge mobilization

#### Knowledge

**Figure 2:**
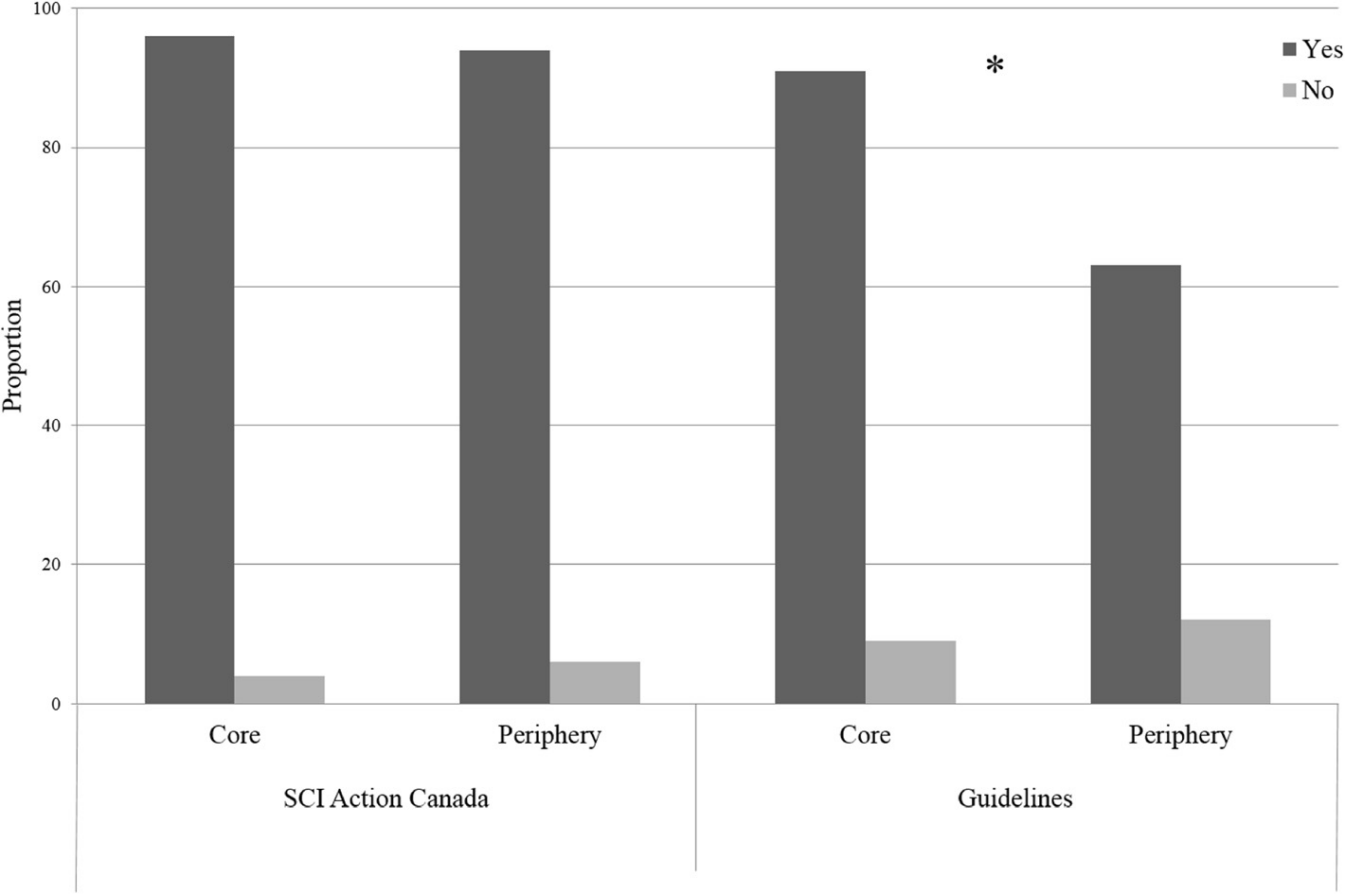
**Association between membership in the core or periphery and knowledge of recommended evidence-based resources.***Note*. * *p* < .05.

Results of the chi square test of independence revealed that membership in the core was associated with knowledge of the physical activity guidelines for people with SCI, *χ*^2^ (1, N = 55) = 0.02, p <0.05. Results of a Fisher’s Exact Test indicated that membership in the core was not associated with knowledge of SCI Action Canada, *χ*^2^ (1, N = 55) = 7.59, p >0.05, (see Figure 2).

#### Behaviour

Results of an ANOVA revealed that members of the core engaged in more behaviours indicative of adoption than members of the periphery, F (1, 33) = 5.34, p = 0.03, d = 0.77 (see Table 1).

**Table 1:** Continuous outcome results

	**ANOVA**		**Point biserial correlation**	
**Outcome**	**Core *Mean (SD)***	**Periphery *Mean (SD)***	**Out-degree centrality**	**In-degree centrality**
***Knowledge***				
SCI Action Canada	--	--	0.06	0.15
SCI Physical Activity Guidelines	--	--	0.11	0.19
***Behaviour***	2.33 (0.91)*	1.47 (1.29)	0.16	0.34*

Note. *p <0.05, SD = standard deviation.

### Degree centrality and knowledge mobilization

#### Knowledge

Point-biserial correlations revealed that neither in-degree centrality nor out-degree centrality were associated with knowledge of SCI Action Canada or the guidelines (see Table 1).

#### Behaviour

Point-biserial correlations revealed that in-degree centrality was significantly associated with adoption behaviours. Out degree centrality was not associated with adoption behaviours (see Table 1).

## Discussion

The present study is the first to examine the association between interpersonal communication and knowledge mobilization within a CBO using network analysis. Consistent with our first hypothesis, membership in the core, as opposed to membership in the periphery, was associated with greater knowledge of the evidence-based physical activity guidelines; and engagement in physical activity promotion behaviours. Our second hypothesis was partially confirmed, higher in-degree centrality was related to greater adoption behaviours. However, greater out-degree centrality was not related to engagement in physical activity promotion behaviours. Neither out-degree nor in-degree centrality were associated with knowledge of the evidence-based physical activity resources. Findings from the present study not only contribute to a small, yet emerging body of literature examining knowledge mobilization within CBOs but also validate and extend the tenets of diffusion of innovations theory using network analysis.

Findings demonstrating that membership in the core as opposed to the periphery is associated with knowledge mobilization align with diffusion of innovations theory. As Rogers’ [13] suggests, information exchange through interpersonal communication pathways is essential for adopting a new practice. The core-periphery structure indicates that individuals in the core had greater opportunities to both receive and disseminate physical activity information than those in the periphery. By sharing information about physical activity, individuals in the core were likely provided with opportunity to discuss, clarify and secure additional information about the evidence-based physical activity materials [13]. Consistent with diffusion of innovations theory, opportunities to exchange information about an innovation likely facilitated the adoption of evidence-based practice among individuals in the core. Conversely, lack of opportunity to exchange information likely hindered adoption among individuals in the periphery. These findings highlight interpersonal communication as an important aspect of the knowledge mobilization process.

Our findings extend Rogers [13] hypothesizing by providing a nuanced understanding of the role interpersonal communication channels in the process of knowledge mobilization. In line with Granovetters’ [34] strength of weak ties theory and Valente *et al.*[21] work, our findings indicate that practitioners should be cautious of dense network structures. Granovetters’ strength of weak ties theory states that less dense network pathways provide links to individuals outside a group or system [34]. Accordingly, Valente *et al.*[21] found that community coalitions with less dense communication structures were more likely to adopt an evidence-based substance abuse prevention program than coalitions with dense networks. Less dense community coalition networks tended to give community leaders access to sources of power and information outside of their group [21,34]. In the present study, the high density of communication pathways observed in the core of the CBO excluded peripheral members of the CBO, thereby, likely stifling opportunities for peripheral members to exchange information and adopt the evidence-based resources. Perhaps the ideal network structure for encouraging knowledge mobilization within a CBO may not be one of high density but one of uniform density in which the core-periphery structure is dissolved and ties are equally developed among all actors in the network.

Finally, our findings also indicate that the effectiveness of communications efforts by individuals in the network are likely dependent on the quality of information being exchanged. Reciprocity within the CBO network is low, indicating that while individuals may have shared information with an individual, the named individual did not confirm the interaction. This finding coupled with findings demonstrating that that in-degree centrality as opposed to out-degree centrality is related to adoption of evidence-based practice highlight the importance of quality of interpersonal communication as opposed to quantity of communication. Individuals with a high out-degree score indicated that they had many interactions where they shared physical activity information. However, out-degree scores were not related to the actual adoption of the evidence-based material. Without adopting the evidence-based practice themselves, these individuals likely did not discuss evidence-based information during their interactions. Conversely, individuals with high in-degree scores were named by others in the CBO as individuals who shared physical activity information. High in-degree scores were related to the adoption of evidence-based materials; therefore, during these interactions these individuals likely discussed evidence-based information. By discussing evidence-based information these individuals may have been perceived as opinion leaders who were credible or prestigious within the network and therefore the interactions were more easily recalled by others in the network [26]. As such, CBOs aiming to mobilize knowledge within the organizations should not assume that all communication efforts are equal. For example, to foster knowledge mobilization, staff in the present network should not only be expected to communicate about physical activity but also have knowledge of evidence-based resources and use these resources in their practice.

### Strengths and limitations

The network analysis approach used in the present study is valuable. To date, the use of diffusion of innovations theory in knowledge mobilization research has required researchers to assume that the diffusion of an innovation is synonymous with the mobilization of knowledge [10]. Our findings address this limitation by empirically validating tenets of diffusion of innovations theory in the context of a CBO. Nevertheless, interpersonal communication is only one aspect of diffusion of innovations theory and a CBO is only one context where knowledge mobilization occurs. As Fujimoto *et al.*[22] findings suggest, networks are dynamic and context specific. Different findings may be observed depending on the context and the network relations being examined. Future research is needed to understand how various network structures, relations and information sharing methods facilitate knowledge mobilization in various settings.

Besides the inherent limitations to network analysis, the design of the present study also has a number of limitations. First, data collection was done using self-report questionnaires which is subject to response and recall bias. Efforts to mitigate these biases were taken by developing our network instrument in partnership with the CBO and testing the face validity of our network instrument (Gainforth HL, Latimer-Cheung AE, Athanasopoulous P, Moore S, Martin Ginis K: Using Network Analysis to Understand Knowledge Mobilization in a Community-based Organization, Submitted). However, the test-retest reliability of our instrument was not assessed. Second, the study used a cross-sectional design which leads to both statistical and practical limitations. Statistically, we cannot determine the directionality of the association between network structure and knowledge mobilization. Practically, a cross-sectional design only allows for a ‘snapshot’ of the CBO at specific time. Networks are constantly evolving. Without conducting a network analysis on a regular basis, the value of the static view of the network is limited [35]. Finally, we did not assess the method participants used to communicate about physical activity (*e.g.*, email, face-to-face). It is possible that different methods of communication were more easily recalled and effective for promoting knowledge mobilization within the CBO.

## Conclusions

Despite these limitations, the present study is the first to formally examine the association of interpersonal communication and knowledge mobilization activities within a CBO. Using a whole network analysis, the present study builds and extends beyond current approaches to examining the process of knowledge mobilization. While further research is needed, results both validate the tenets of diffusion of innovations theory and highlight the importance of fostering opportunities for interpersonal communication in the process of knowledge mobilization.

## Endnote

^a^Volunteers within the CBO also were invited to complete the network questionnaire. However, volunteers were omitted from the analysis due to a low response rate (9%). Of note, a core-periphery network structure was evident in exploratory analyses that included volunteers.

## Abbreviations

CBO: Community-Based Organization; CIHR: Canadian Institutes of Health Research; SCI: Spinal Cord Injury; SSHRC: Social Sciences and Humanities Research Council of Canada.

## Competing interests

The authors declare that they have no competing interests.

## Authors’ contributions

HG devised the research question and was responsible for developing the network instrument and supplementary materials; instructing participants about how to complete the instrument; collecting, entering and analyzing data; and writing the manuscript. ALC provided input with regards to the design; statistical analyses; interpretation of results; and editorial feedback on the manuscript. SM provided input regarding the design; network and statistical analysis; interpretation of results; and editorial feedback. PA and KMG provided input regarding study design and editorial feedback. PA and KMG established the partnership that allowed for the research to be conducted in the CBO. All authors read and approved the final manuscript.
